# EBV and the Pathogenesis of NK/T Cell Lymphoma

**DOI:** 10.3390/cancers13061414

**Published:** 2021-03-19

**Authors:** Ivonne A. Montes-Mojarro, Falko Fend, Leticia Quintanilla-Martinez

**Affiliations:** Institute of Pathology and Neuropathology and Comprehensive Cancer Center Tübingen, Eberhard-Karls-University, 72076 Tübingen, Germany; Ivonne.Montes@med.uni-tuebingen.de (I.A.M.-M.); Falko.Fend@med.uni-tuebingen.de (F.F.)

**Keywords:** Epstein-Barr virus, strain, pathogenesis, epidemiology, genetic landscape

## Abstract

**Simple Summary:**

Extranodal NK/T cell lymphoma (ENKTCL) is an aggressive lymphoma associated with Epstein-Barr virus (EBV) infection that occurs mainly in Asian and Latin American populations. In the last decade, the genetic landscape of ENKTCL has been characterized comprehensively using next-generation sequencing (NGS). This and similar high-throughput approaches revealed that these lymphomas are distinguished by frequent gene mutations leading to activation of the JAK-STAT pathway, and mutations in other genes such as *BCOR, DDX3X* and *TP53*. This review aims to provide a comprehensive overview about the role of EBV infection and a comparison of the EBV strains and LMP1 variants among different populations. Moreover, a brief summary of the ENKTCL genetic landscape is presented, highlighting the main therapeutically targetable pathways in ENKTCL oncogenesis: the JAK-STAT signaling pathway, the immune response evasion, *MYC* overexpression, as well as epigenetic alterations.

**Abstract:**

Epstein-Barr virus (EBV) is a ubiquitous gamma herpes virus with tropism for B cells. EBV is linked to the pathogenesis of B cell, T cell and NK cell lymphoproliferations, with extranodal NK/T cell lymphoma, nasal type (ENKTCL) being the prototype of an EBV-driven lymphoma. ENKTCL is an aggressive neoplasm, particularly widespread in East Asia and the native population of Latin America, which suggests a strong genetic predisposition. The link between ENKTCL and different populations has been partially explored. EBV genome sequencing analysis recognized two types of strains and identified variants of the latent membrane protein 1 (LMP1), which revealed different oncogenic potential. In general, most ENKTCL patients carry EBV type A with LMP1 wild type, although the LMP1 variant with a 30 base pair deletion is also common, especially in the EBV type B, where it is necessary for oncogenic transformation. Contemporary high-throughput mutational analyses have discovered recurrent gene mutations leading to activation of the JAK-STAT pathway, and mutations in other genes such as *BCOR, DDX3X* and *TP53*. The genomic landscape in ENKTCL highlights mechanisms of lymphomagenesis, such as immune response evasion, secondary to alterations in signaling pathways or epigenetics that directly or indirectly interfere with oncogenes or tumor suppressor genes. This overview discusses the most important findings of EBV pathogenesis and genetics in ENKTCL.

## 1. Introduction

Extranodal NK/T cell lymphoma, nasal type (ENKTCL) is considered the prototype of EBV-driven T and NK cell lymphoproliferative disorder (LPD). It has a predilection for extranodal involvement, including the nasopharyngeal region, skin, gastrointestinal tract, testis, and central nervous system (CNS). Morphologically, ENKTCL is characterized by angioinvasion and angiodestruction with prominent coagulative necrosis and karyorrhexis [1]. Its pathogenesis is still unclear, but the characteristic geographical distribution suggests that ethnicity plays an important role [1,2]. ENKTCL predominantly occurs in East Asia and in the indigenous populations of Latin American countries, and it is rare in Europe and North America [3,4,5,6,7,8]. Other diseases with similar geographic distribution and universal association with EBV are aggressive NK cell leukemia (ANKL) and the provisional entity *primary EBV-positive nodal T cell or NK cell lymphoma*. There are some clinical and morphological overlapping features among these entities, therefore differential diagnosis is not always easy [9,10,11,12,13].

The contribution of EBV to the development of benign and malignant LPD has been extensively investigated since the discovery of the virus over the last 60 years. The importance of EBV in ENKTCL lymphomagenesis was first recognized in 1990, and since then has been confirmed in various studies [14,15]. To investigate the role of EBV in the pathogenesis of this lymphoma, further studies have focused on the viral proteins and their genetic variants [16,17]. Moreover, the strong geographic distribution of ENKTCL affecting specific populations suggests a genetic predisposition for ENKTCL. So far, common genetic variants at HLA-DPB1 in patients from Asia (Hong Kong, Taiwan, Singapore, and South Korea) are associated with an increased risk of ENKTCL [18], whereas the haplotype HLA-A*0201 in the Japanese population seems to confer protection, probably by an effective cellular immune response against the virus [19]. There has also been a great interest in the mutational landscape of ENKTCL. Until now, most studies come from Asian countries, where important variations in the distribution of gene mutations were observed, raising the possibility that the genetic background or the geographic distribution might be responsible for these differences [20,21,22,23,24]. This review summarizes the morphological, epidemiological, biological, and genetic alterations implicated in ENKTCL, highlighting the role of EBV in its pathogenesis. In addition, a comparison of the biological features of ENKTCL between Asian and Latin American populations is described. 

## 2. Morphological and Immunophenotypical Features of ENKTCL 

ENKTCL typically appears as ulceration in the mucosa of the upper aero-digestive tract (nasal cavity, nasopharynx, paranasal sinuses, and palate). This ulceration is triggered by a neoplastic lymphoid infiltration associated with variable degrees of inflammation; some cases are advanced, showing large amounts of coagulative necrosis and destruction of the adjacent epithelial structures [1,25,26]. The cytology of the malignant T and NK cells displays a wide-spectrum, from small bland-looking cells to large and pleomorphic cells with irregular folded nuclei and inconspicuous nucleoli [1]. The presence of “dirty” coagulative necrosis, due to karyorrhexis, associated with inflammation and angiocentricity/angiodestruction is a hallmark of this lymphoma and a hint for EBV infection that should be confirmed using in situ hybridization for EBV-encoded small RNA (EBER) [1]. The neoplastic cells are CD56+, surface CD3− but cytoplasmic CD3+, and express cytotoxic molecules such as TIA-1, granzyme B and perforin, demonstrating an NK cell phenotype. In addition, the cells are positive for CD2, NKG2D and NKG2A; however, CD57 and CD16 remain mostly negative [27,28]. Other T cell markers such as CD4, CD5 and CD8 are negative whereas CD7 is variable expressed [29,30]. A small proportion of cases (15–20%) demonstrate a *bona-fide* T cell cytotoxic phenotype characterized by CD8+, cytotoxic granules+, CD3+, CD5+, CD56−/+ and TCRγδ+ or TCRαβ+ [29,30]. In addition, molecules with immune response function are present in the neoplastic cells such as CD25, HLA-DR, FAS (CD95) and FASL (CD95L) [31]. CD30 and the megakaryocyte associated tyrosine kinase (MATK) are variably expressed and might be misleading, raising the diagnosis of anaplastic large cell lymphoma (ALCL) or monomorphic epitheliotropic intestinal T cell lymphoma (MEITL) and enteropathy associated T cell lymphoma (EITL) [32,33]. Other key markers recognized as potential targets for therapy are survivin, as well as the platelet-derived growth factor receptor alpha (PDGFRA) and the programmed cell death ligand 1 (PD-L1), the last two involved in immune evasion mechanisms [34,35]. 

## 3. ENKTCL Geographic Distribution

The geographic distribution of ENKTCL is characteristic with higher incidence among Native Americans, Hispanic and Asian ethnic groups [36]. In Europe and North America ENKTCL accounts for less than 1% of all Non-Hodgkin Lymphomas (NHL), whereas in Asian countries and Latin American countries it is more frequent representing 2 to 15% of NHL [3,4,5,6]. A recent study from the T cell lymphoma project, an international cooperative study, demonstrated NK cell malignant lymphoproliferations accounted for 2 to 5.1% of all T and NK cell lymphomas in North America, 1 to 4.3% in Europeans, and higher rates in Asian populations of 22.4 to 22.5% [37]. Aozasa et al. estimated that the frequency of ENKTCL is 10-fold higher in Asian populations when compared to Europeans [38]. In East Asia, ENKTCL is more common in Thailand (34%), followed by China (21%), Japan (12%), and South Korea (9%) [39]. Moreover, ENKTCL is strongly associated with the genetic background of the affected population, being more frequent in Chinese descendants than those with Malay and Indian descent [40,41]. In Latin America, the incidence of ENKTCL is higher in countries with a high proportion of native indigenous population such as Guatemala, Mexico, Peru, Bolivia and Ecuador; it is less common in other countries with a greater percentage of European descendants such as Argentina and Uruguay [8]. Fourteen different series of ENKTCL are available from Latin American, accounting 449 cases: four studies with cases from Peru (131 cases, 29.2%) [7,42,43,44], four studies from Brazil (114 cases, 25.4%) [7,45,46,47], two studies from Guatemala (125 cases, 27.8%) [7,48], two from Chile (31 cases, 6.9%) [47,49], and two from Mexico (48 cases, 10.7%) [6,50].

An ENKTCL case series from Guatemala described the association of this lymphoma with ethnicity and reported that 90% of the patients in the study were of Mayan origin and low socioeconomic status [51]. In the United States, ENKTCL represents approximately 1–2% of all T and NK cell lymphomas and less than 0.2% of all NHL, with a higher frequency among Hispanic and American Asians [52].

**Figure 1:**
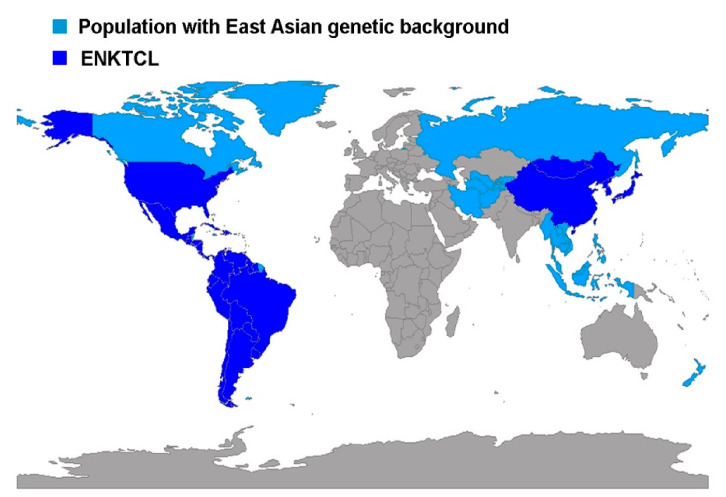
Map illustrating the worldwide distribution of extranodal NK/T cell lymphoma (ENKTCL) in countries with a population of east Asian genetic background. East Asian populations are depicted in blue; countries with high prevalence of ENKTCL are illustrated in dark blue. This map was elaborated using R version 3,6,2 with the package rworldmap [56].

A bias in these studies is that Hispanic population comprises all Latin-American immigrants, mainly Mexicans, which do not necessarily belong to the native indigenous population, where the incidence of this neoplasia seems to be higher [51,52]. The peculiar worldwide distribution of this lymphoma seems to be related with the genetic background of the population (Figure 1), and not to endemic areas of EBV or EBV subtypes [53]. Nevertheless, evidence for the suspected genetic predisposition such as the HLA-DBP1 haplotype remain elusive [18]. Homozygous deletion of *RASGRPI,* leading to defective activation of the MAPK pathway and impaired immune response to EBV, is also documented as an inherited susceptibility to EBV infection and EBV-driven LPD such as ENKTCL [54]. In addition, homozygous germline mutation in FAM160A1 leading to alterations in the microenvironment is related to ENKTCL familiar susceptibility [55].

## 4. Epstein-Barr Virus Lymphomagenesis in ENKTCL

Although the universal association of EBV and ENKTCL is well recognized, the exact role of EBV in ENKTCL remains elusive [2]. EBV is an oncogenic double-stranded DNA herpesvirus encoding around 80–85 genes, infecting more than 90% of the population worldwide [57]. In developing countries, EBV infection is more common during the first years of life and is asymptomatic, whereas in developed countries, it is delayed until adolescence, presenting as a self-limited B cell lymphoproliferative disease named by Sprunt and Evans in 1920 as infectious mononucleosis (IM) [58,59,60]. IM is an acute disease occurring in about 50% of adolescents with the characteristic symptoms of fever, fatigue, sore throat, and lymphadenopathy [61,62]. During primary infection, EBV triggers an EBV-specific cytotoxic cell and IgM response to EBV antigens such as EBV capsid antigen (VCA) and EBV early antigen (EA) [63]. This response leads to a self-limited infection; nevertheless, EBV remains in a lifelong carrier state mainly in memory B cells, allowing the DNA virus to integrate into the host cells and reside there life-long. During this chronic infection, various external or immune factors may incite the infected cells to enter the viral lytic cycle, triggering their activation and the virus transmission to T cells or NK cells and stimulating the development of different lymphoproliferative disorders [64,65,66,67]. EBV is detected in a small proportion of NK and T cells in patients with IM. During primary infection, NK cells are infected using a virus–cell interaction distinct from the CD21-mediated pathway known in B cells. One of the mechanisms proposed is related to the use of the glycoprotein gp350, as well as the CD21 cellular protein gaining in order to infect the NK and/or mature T cells via “trogocytosis”, a phenomenon described in the interaction of mature T cells with malignant cells, where membrane patches can be exchanged [68,69]. Another mechanism of T and NK cell infection can occur when NK or T cells are attempting to kill an EBV infected target cell [69]. The expression of HLA class II by NK cells has also been proposed to interact with viral glycoproteins gp42 and gp85 known to play an important role in EBV internalization into HLA class II positive cells [70]. 

EBV expresses various “latent genes”, comprising six Epstein-Barr nuclear antigens (EBNA1, EBNA2, EBNA3A, EBNA3B, EBNA3C and leader protein), three latent membrane proteins (LMP1, 2A, 2B), two EBV-encoded noncoding RNAs (EBER 1 and 2), and many miRNAs from two regions of the EBV genome: BART and BHRF, so-called BHRF1- and BART-miR [71]. According to the pattern of the latent gene expression, three different latency programs have been characterized in B cells from EBV healthy carriers [72,73,74]. EBV characteristically spreads through saliva, infecting and replicating in epithelial cells of the tonsils (lytic cycle), states in which the viral transcription factors *BZLF1, BRLF1, BALF5* and *BCRF1* are expressed, resulting in the shedding of infectious virus into the oral cavity [75,76]. Infected naïve B cells migrate to the lymph node follicle to initiate the immune response activating the latency III program, which involves the unrestricted expression of all nine latent genes. In order to escape immune surveillance, and for the infected B cells to be able to enter and survive the germinal center, there is a reduction in the latent gene transcription machinery–latency II—with the expression of many proteins (LMP1+, LMP2+; EBNA1+, EBERs+, BARTs+), except for EBNA2. Latency I is restricted to the expression of EBNA1 and is responsible for the maintenance and replication of the episomal EBV genome [72,73,74]. The so-called latency 0 is present in resting memory B cells that carry the viral genome, but viral antigen expression is maximally suppressed and only EBERs are demonstrated [73,77,78]. These latent EBV expression programs in B cells are reflected in EBV-associated malignancies. Latency type I is usually present in Burkitt Lymphoma; latency IIa, also referred to as latency II, is seen in nasopharyngeal carcinoma, classic Hodgkin lymphoma (CHL) and ENKTCL [72,74,79]; latency IIb represents a transition state between latency II and latency III, which is detected in vitro in cells from B cell chronic lymphocytic leukemia (B-CLL) infected with EBV, characterized by EBNAs expression but LMP1 absence [72], and latency III is typically observed in severely immunodeficient individuals [80]. During latency II, the infected cells can acquire somatic mutations and create the best scenario to promote the development of EBV-associated lymphomas such CHL and Burkitt lymphoma [72,78].

### 4.1. Epstein-Barr Virus Strains and Variations

The close association of EBV with oncogenesis in ENKTCL has been established by demonstrating the presence of EBV in clonal and episomal forms in tumor cells (viral genome arranged by nucleosomes and packaged into a chromosome structure), as well as various EBV-encoded proteins. Infection by EBV induces the expression of several “latent genes”, which may lead to malignant transformation of lymphoblastoid cells, including EBNA1, EBNA2, EBNA3A, EBNA3B, EBNA3C and 3 LMP1, 2A, 2B [71]. According to the genetic polymorphisms contained in the EBNA proteins (EBNA2 and EBNA-3A, -3B and -3C), two different strains of EBV are recognized worldwide (Table 1): type A (e.g., B95-8, GD1, and Akata) and type B (e.g., AG876 and P3HR), also known as type 1 and type 2, respectively [16,17]. 

Sequencing studies have shown a 54% identity in amino acid sequence and 79% at gene level between EBV strains type A and B. In addition, a single amino acid change (S442D) in EBV type A seems to enhance the oncogenic ability of LMP1 [17], highlighting that EBV strain type A has a higher oncogenic potential in comparison to type B EBV, which shows lower transformation capacity in lymphoblastoid cells [87]. EBV type B is widely distributed, regardless of the immunological status of the host [88,89,90,91,92]; however, in the setting of non-immunocompromised patients, EBV type B seems to enhance its oncogenic potential only when associated to the 30 bp LMP1 deletion (see below) [43,87,93]. Intriguingly, these strains demonstrate a characteristic geographical distribution that might influence the ENKTCL prevalence among regions. Although type A EBV strain is more widespread in Europe, Asia, and North and Latin America, type B EBV is recurrently seen in Alaska, Papua New Guinea, and Central Africa [17].

Interestingly, studies in Mexican population have revealed the presence of EBV type B with the 30 bp LMP1 deletion in about 9% of ENKTCL, 38% in diffuse large B cell lymphoma (DLBCL), 50% of CHL, and 53% in healthy carriers in reactive lymph nodes [82,94,95]. This suggests that EBV type B with the 30 bp LMP1 deletion is endemic in the Mexican population. However, larger ENKTCL series from Mexico and Latin America demonstrated EBV strain type A as the most prevalent strain in this lymphoma [6,82]. In addition to the classification of the EBV strain, other variants according to changes within the genetic sequence of LMP1, EBNA1 and BamHI are also reported [96,97,98,99,100,101]. 

### 4.2. LMP1 Variants

The latent membrane protein 1 (LMP1) is a viral protein that is able to induce a malignant transformation not only in B cells but also in epithelial cells [97,102,103]. LMP1 favors oncogenesis by the induction of cell surface adhesion molecules (CD23, CD40 ICAM1, LAF1 and LFA3), activation of antigens, and the upregulation of antiapoptotic molecules such as BCL2, MCL1, BFL1, A20 [75,103]. Moreover, LMP1 mimics CD40, acting as a constitutively active member of the tumor necrosis factor (TNF) receptor superfamily and activates downstream signaling pathways, including NF-κB and MAPK pathways [104,105,106]. LMP1 is codified by *BNLF1,* a gene located within the BamHI-N region of the virus genome [107]. The product of this gene is an integral protein which contains 386 amino acids comprising a short cytoplasmic amino terminus, six transmembrane alpha-helical loops of hydrophobic nature, and a long cytoplasmic domain at the carboxyl terminus [104,108]. Variations in the C-terminus of the LMP1 protein seem to be crucial for its function, and for the classification of LMP1 variants, including: the presence of a 30 bp deletion, 33 bp repeats, an insertion of 15 bp within one of the repeats, and other amino acid substitutions [98,109,110,111]. Among the different LMP1 variants, the 30 bp deletion is the most frequent worldwide [112]. The 30 bp deletion arises at the 3′ end of the C-terminal tail and in relation to the functional domain CTAR2, resulting in increased oncogenesis and a decrease in the immune response [113,114]. LMP1 deletion is present in healthy populations, as well as associated to infectious mononucleosis, chronic tonsillar hyperplasia, and various malignant neoplasias such as gastric carcinoma, nasopharyngeal carcinoma, Burkitt lymphoma, DLBCL, and CHL peripheral T cell lymphomas, not otherwise specified (PTCL, NOS) and ENKTCL (Table 2) [86,95,112,115,116,117]. 

Next-generation sequencing technology has also led to greater insights into EBV classification and the increased recognition of new strains and sequence variations [17,90]. EBV sequencing profiling revealed frequent intragenic deletions affecting BART micro-RNA clusters present in 10 of 23 ENKTCL cases studied. These deletions seem to be related to the lytic cycle activation through the upregulation of *BZLF1* and *BRLF1* [118].

The latest genomic and transcriptomic studies in ENKTCL demonstrated focal EBV genome deletions and integration of EBV fragments to the host genome. In addition, gene expression profiling described a higher number of T cell epitope abnormalities but lower activation of latent and lytic viral genes, in comparison to other EBV-associated cancers [119]. Likewise, by phylogenetic analysis, EBV sequences of ENKCTL cases clustered together in two independent Chinese studies, revealing similarities between EBV sequences in ENKTCL and those of Asia, and differed significantly from other EBV-associated diseases. These results suggest that the geographic prevalence of ENKTCL in Asian populations is related to the particular sequence of the EBV strain [81,119]. These broad genetic analyses have revealed a better understanding of EBV pathogenesis in ENKTCL and are providing new hypotheses about EBV mechanisms of oncogenesis. However, the majority of cases are from Asian origins, and larger series are required to corroborate these findings among other populations [119].

**Table 2:** Geographical distribution of LMP1 variants in T cell non-Hodgkin lymphoma.

Country	Entity	*n*	LMP1 Variant		Reference
			30 bp del	WT	
Mexico	ENKTCL	42 cases	10 (23.8%)	32 (76.2%)	[82]
Peru	ENKTCL	27 cases	0	12 (100%)	[78]
Argentina	ENKTCL	12 cases	5 (41.7%)	7 (58.3%)	[82]
China	ENKTCL	13 cases	10 (76.9%)	3 (23.1%)	[120]
China	ENKTCL	23 cases	21 (91.3%)	2 (8.7%)	[84]
Mexico	ENKTCL	23 cases	6 (26%)	17 (73.9%)	[6]
Malaysia	PTCL	9 cases	9 (100%)	0	[86]
Denmark	PTCL	18 cases	11 (61.1%)	7 (38.9%)	[86]

ENKTCL: extranodal natural killer T cell lymphoma; 30 bp del: 30 base pair deletion variant; WT: wild type; PTCL: peripheral T cell lymphoma. In addition to the LMP1 del variant, the loss of the restriction site *Xho I* in LMP1 has been shown in nasopharyngeal carcinoma in Asian pop-ulation [97,121,122,123].

## 5. ENKTCL Genetic Features

Although EBV plays an essential role in the pathogenesis of ENKTCL, other factors might be equally important. During the last decade, several sequencing studies based on various platforms (whole genome sequencing, whole exome sequencing and targeted sequencing) have been published, analyzing different populations. This has led to a better understanding of the molecular pathogenesis of this entity, the discovery of new targeted therapies, and more recently, to the proposal of a classification according to the molecular changes associated with clinical prognosis. In Table 3, the main genetic features reported in ENKTCL so far are summarized [81,124,125]. 

The most common recurrent cytogenetic alteration in ENKTCL is the loss of 6q21 in about 20 to 43% of cases, resulting in the loss of genes related to tumor suppression such as *POPDC3, PREP, PRDM1, ATG5, AIM1* and *HACE1* [126,127,128]. Other recurrent chromosomal alterations are losses in chromosomes 1p4, 5p13, 12q3, 14q21, 15q24, 17p4 and 19q13 and gains in 2q5, 3q26, 7q34, 8q24, 13q4 and 10q3. Intriguingly, 8q24.3 gain is related to poor clinical outcome [129,130].

Further analyses have shown important differences between ENKTCL and ANKL, such as the gain of 2q and the losses in chromosomes 6q16-q27, 11q22-q23, 5p14-p14, 5q34-q35, 1p36-p36, 2p16, 4q12, and 4q31-q32 characteristic in ENKTCL [131,132]. Studies have demonstrated that *primary EBV-positive nodal T/NK cell lymphoma*, a provisional entity in the WHO classification, is a distinct entity characterized by the loss of 14q11, where the TCR alfa locus is located and is indirect evidence of TCR rearrangement; therefore, it is a lymphoma of T cell origin. These lymphomas present in older patients and lack nasal or extranodal involvement [133]. 

**Figure 2:**
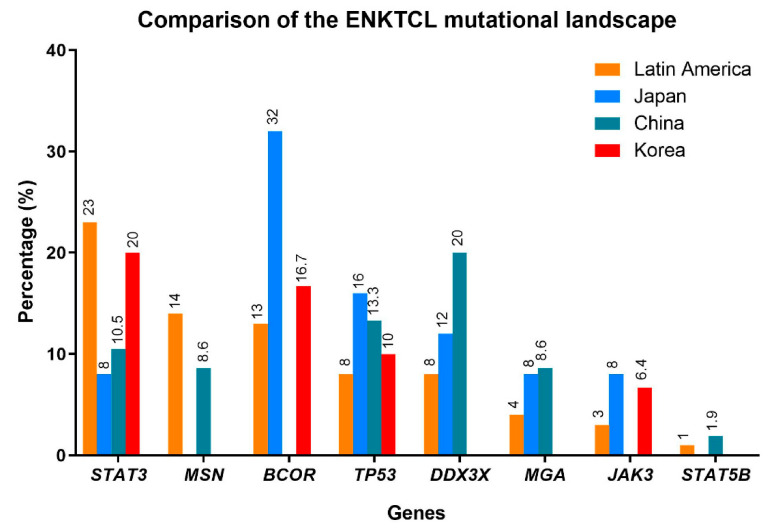
Comparison of the ENKTCL mutation landscape in different populations. The frequency of the mutations of the most common altered genes among these populations is depicted as percentages. The colors indicate the different populations in four different studies: Latin America (orange) [82], Japan (blue) [23], China (green) [22], and Korea (red) [20].

The mutational landscape of ENKTCL was first described in different cohorts from China, Korea and Japan, showing the same mutational profile but with different frequencies. The most recurrently mutated genes include members of the Janus kinase-signal transducer and activator of transcription (JAK-STAT) signaling pathway, mainly *STAT3* (*JAK3, STAT5B*), followed by epigenetic modifiers (*KMT2D*, *ARID1A, EP300*), tumor suppressor genes (*TP53, BCOR, MGA*) and the RNA helicase gene *DDX3X* [20,21,22,23,24,135]. Targeted sequencing in ENKTCL from Latin America including cases from Mexico, Peru and Argentina showed, not surprisingly, comparable results to Asian populations (Figure 2) [82]. *STAT3* was the most frequently mutated gene similar to the Korean cohorts. [20,125,135]. In previous studies, recurrent mutations in *TP53* were reported with relatively high frequencies in ENKTCL from Latin America [50] and in Asian populations [156,157], (24 to 62%, respectively); however, new sequencing data demonstrated a lower prevalence of *TP53* mutations in the Chinese [21] and in the Latin American studies [82]. *DDX3X*, another tumor suppressor, is also frequently mutated in the Chinese population and strongly associated with poor outcome [21], not confirmed in other studies [20,21,22,23,24]. The different frequencies reported in the different populations might be the result of the various analytic methods used (whole-exome sequencing, targeted sequencing, RNA-sequencing) in the different studies more than representing real differences in the genetic background or geographic distribution. 

Epigenetic alterations such as methylation in cell cycle regulators, histone modifications, and de-regulated miRNAs are also described. Aberrant promoter methylation is a common oncogenic mechanism in ENKTCL, which induces the silencing of various tumor suppressor genes such as *BCL2L11 (BIM), DAPK1, PTPN6 (SHP1), TET2, SOCS6,* and *ASNS* [141], as well as in regulators of the cell cycle such as *CDKN2A, CDKN2B*, and *CDKN1A* [140]. *EZH2* is a histone methyltransferase, a member of the polycomb repressor complex 2 (PCR2) which controls the DNA methylation of oncogenes and tumor suppressor genes acting as a transcriptional repressor. In ENKTCL, *EZH2* not only acts as a repressor, but also acts as an oncogene, promoting proliferation, invasion and survival through the NF-kB signaling pathway [142,143,144]. Further key epigenetic regulators are miRNA, small non-coding RNAs capable of inducing post-transcriptional gene regulation acting as oncogenes or as tumor suppressor genes [158,159,160]. Numerous deregulated miRNA have been identified in ENKTCL, including the downregulated miR-146a, miR-26a, miR-26b, miR-28-5, miR-101 and miR-363 [144,145], whereas miR21 and miR155 are upregulated probably acting as oncogenes, stimulating proliferation through the AKT and MAPK signaling pathways [137,146]. On the other hand, overexpression of the tumor suppressor miR-146a in vitro leads to downregulation of the NF-kB signaling pathway by the inhibition of its target *TRAF6,* resulting in repressed cell proliferation, induced apoptosis, and enhanced chemosensitivity [145]. The profiling and biogenesis of miRNAs has been studied in various disorders and not only in ENKTCL, because they can be exploited and used as biomarkers, but also as new therapeutic target by inhibiting miRNA oncogenes or by the miRNA replacement of tumor suppressor miRNAs [161]. In the last decade, miRNA therapies for cancer, metabolic and infectious diseases have reached the preclinical phase of commercial development, representing a new therapy approach in these disorders [161,162,163].

Gene expression profiling has also enabled the distinguishing of upregulated genes, with an important role in the pathogenesis of ENKTCL including *BIRC5* encoding for survivin, *MYC, PD-L1, RUNX3, AURKA*, and *PDGFRA*. *MYC* is a well-known oncogene related to aggressive clinical behavior in mature B cell lymphomas [164], and is associated with *NOTCH1* mutations in T cell lymphomas [165]. Overexpression of MYC in ENKTCL seems to be related to alterations in the MAPK signaling pathway and the EBV pathogenesis because MYC is a transcriptional target of EBNA1 and LMP1 viral proteins [144]. Interestingly, *MGA* mutations and 1p22.1 loss of heterozygosity are similarly related to MYC expression promoting the disturbance of the MAPK signaling pathway [81].

## 6. ENKTCL Proposed Molecular Classification

**Figure 3:**
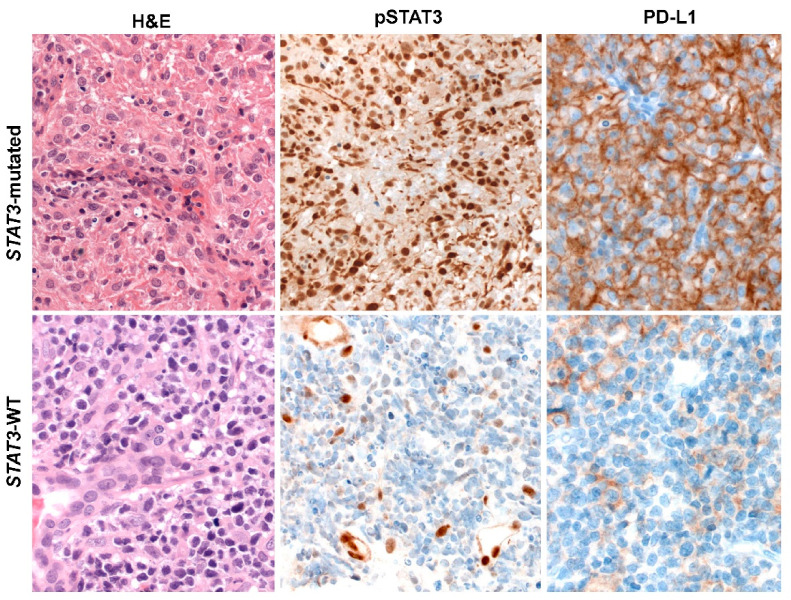
JAK-STAT signaling pathway and immune response evasion disturbances in ENKTCL. NKTCL biopsy carrying *STAT3* activating mutation (c.1919A>T, p. Y640F) belonging to the tumor suppressors and immune modulators (TSIM) molecular group. Large-size tumor cells in a necrosis background are depicted (H&E) positive for pSTAT 3 and PD-L1. ENKTCL biopsy revealing *STAT3* wild type. Neoplastic cells lacking an inflammatory background (H&E). In addition, P-STAT3 is negative in the tumor cells but positive in the vessels as an internal control, whereas PD-L1 is similarly negative in most of tumor cells but positive in some reactive histiocytes (H&E stain and Immunohistochemistry 400×). Abbreviations: H&E, hematoxylin and eosin; EBER, in situ hybridization for EBV-encoded small RNA.

**Figure 4:**
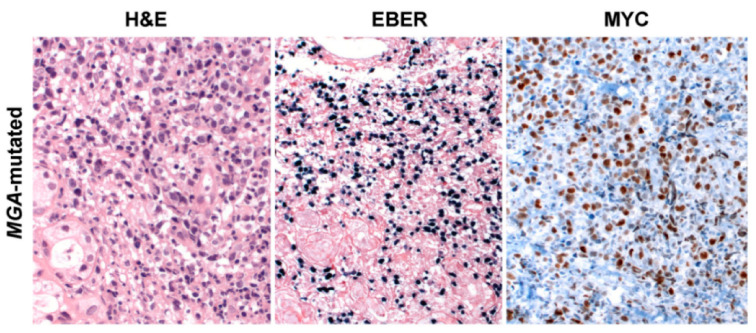
ENKTCL harboring *MGA* frameshift mutation (c.7586 G > A, p.H533*) associated with MYC overexpression belonging to the MB molecular group. ENKTCL with large cell morphology, cells are pleomorphic with pale cytoplasm, and irregular nuclei (H&E stain, 400×); all lymphoma cells show EBER positivity (in situ hybridization 200×) and MYC nuclear expression (immunohistochemistry 400×). Abbreviations: H&E, hematoxylin and eosin; EBER, in situ hybridization for EBV-encoded small RNA.

**Figure 5:**
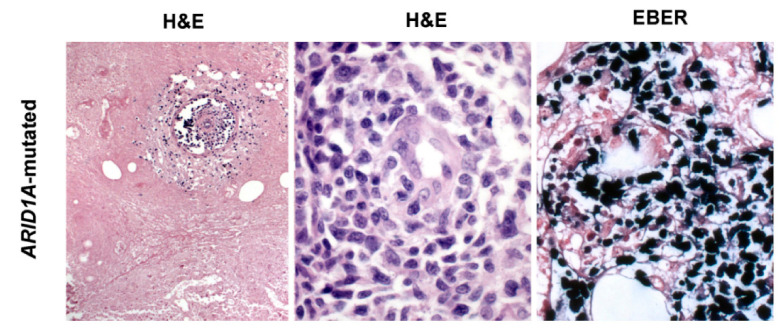
ENKTCL harboring *ARID1A* mutation probably belonging to the HEA molecular group. The morphological hallmark features of ENKTCL are present: coagulative necrosis and large-size pleomorphic neoplastic cells leading to angioinvasion (H&E stain, 200× and 400×). All lymphoma cells show EBER positivity (in situ hybridization, 400×). This case carried also a *DDX3X* missense mutation (c.1537G>C, p. V513L). Abbreviations: H&E, hematoxylin and eosin; EBER, in situ hybridization for EBV-encoded small RNA.

Based on molecular integrated analysis of ENKTCL, a new molecular classification has been proposed, distinguishing three different ENKCTL subtypes defined according to their genetic characteristics [81]. The first group—TSIM subtype (alteration in Tumor Suppressors and Immune Modulators)—consists of cases with JAK-STAT signaling pathway activation, *TP53* mutations, del6q21, and mechanisms associated with the immune response such as antigen presentation, NK cell-mediated cytotoxicity, immune surveillance, PD-L1 overexpression, and genomic instability. This group predominates and presents higher NK cell gene expression than the other groups (Figure 3). The second group—MB subtype (*MGA* mutations and LOH at the *BRDT* locus)—is characterized by *MGA* mutations, a tumor suppressor gene, related to MYC overexpression and associated with MAPK, NOTCH3/4 and WNT signaling pathway activation (Figure 4). *BRDT* enhances oncogenic functions of cancer drivers, including *MYC*. These findings provided evidence that MYC is critically involved in this subtype. The third group—HEA subtype (mutations in *HDAC1, EP300* and *ARID1A*)—is linked to epigenetic changes, mainly aberrant histone acetylation leading to activation of the NF-kB and TCR signaling pathways (*CD3D/G, CD8A/B, CD28, ICOS*, and *VAV2/3*) (Figure 5). These cases show higher T cell gene expression compared to the other groups. These data suggested that the molecular subtypes correlate well with cells of origin (NK vs T cell). Furthermore, it also showed correlation with the predicted three-year overall survival (79%, 39% and 92%, respectively). The worse prognosis observed in the MB subtype confirmed previous observations that MYC overexpression correlates with disease progression and dismal prognosis in ENKTCL [166]. Interestingly, EBV gene transcript levels were differentially expressed in these three molecular subtypes. The TSMI subtype was associated with the expression of EBV lytic genes such as *BALF3*, which increases genomic instability, regulates *TP53* targeted genes, and leads to malignant transformation. The MB subtype showed low expression of EBV genes, particularly LMP1, suggesting that this group could have BL-like EBV infection (latency type I). The HEA subtype was characterized by increased lytic gene *BNFR1*, a partner of the histone chaperon complex, which interacts with DAXX to promote viral latency and cellular immortalization.

## 7. Conclusions

The contemporary understanding of ENKTCL pathogenesis has been enhanced. The use of high-throughput technologies has allowed the genetic analysis of large series of ENKTCL in different populations around the world. These studies have been conducted to investigate the pathogenesis of ENKTCL and the role of EBV in its oncogenesis. EBV strain distribution in ENKTCL shows EBV type A in Asian and Latin American population, whereas type B is identified in Latin American only in Peru and Mexico in comparison to other Western countries. Interestingly, LMP1 30 bp del variant, which promotes EBV oncogenic mechanisms, has been frequently demonstrated in the Chinese population and less commonly in Latin American countries, where its detection is mostly restricted to patients carrying EBV strain type B. 

As has been proposed in diffuse large B cell lymphoma, the integration of genomic structural alterations in ENKTCL has identified three molecular subgroups related to the cell of origin (NK vs T cell), pathogenic alterations, EBV sequences and clinical outcome. The model suggests that EBV infection is the earliest event, leading to the susceptibility of NK and T cells to genomic alterations that together will result in the pathogenesis of ENKTCL. Consequently, these alterations will influence the prognosis and therapy response. The understanding of the genetic alterations and their altered mechanisms should guide novel therapeutic interventions such as immune checkpoint inhibitors, MYC inhibitors, and histone deacetylase inhibitors. Nevertheless, these studies have only been conducted in Chinese populations, and new comprehensive genomics studies are needed to confirm these results. 

## Figures and Tables

**Table 1:** Geographical distribution of Epstein-Barr virus (EBV) strains in T cell non-Hodgkin lymphoma.

Country	Entity	*n*	EBV Strain		Reference
			Type A	Type B	
China	ENKTCL	31 cases	29 (93.5%)	2 (6.5%)	[81]
Mexico	ENKTCL	42 cases	39 (93%)	3 (7%)	[82]
Peru	ENKTCL	27 cases	15 (88%)	3 (12%)	[82]
Argentina	ENKTCL	12 cases	11 (92%)	1 (8%)	[82]
Korea	T cell NHL	15 cases	14 (93.3%)	1 (6.7%)	[83]
China	ENKTCL	16 cases	16 (100%)	0	[84]
Mexico	ENKTCL	23 cases	21 (91%)	2 (9%)	[6]
China/Taiwan	Nasal and extranasal PTCL	19 cases	19 (100%)	1 (5.3%)	[85]
Malaysia	PTCL	9 cases	9 (100%)	0	[86]
Denmark	PTCL	18 cases	15 (83.3%)	3 (16.7%)	[86]

ENKTCL: extranodal natural killer T cell lymphoma; *n*: number of cases reported; PTCL: peripheral T cell lymphomas; NHL: non-Hodgkin lymphoma.

**Table 3:** Main genetic alterations in ENKTCL.

Genetic Alteration				Reference
Chromosomal abnormalities	Losses of 6q21–6q25 (40–50%)	*POPDC3, PREP, PRDM1, ATG5, AIM1 and HACE1*		[126,127]
	Other chromosomal alterations	Losses in 5p13, 11q22-q23,11q24-25, 12q3, 13q14, 14q21, 15q24, 17p13, 17p4 and 19q13 Gains in 1q21-q44, 2q13-14, 2q31-q32, 2q5, 3q26, 6p25-p11, 7q34, 7q35-q36, 8q24, 10q3, 13q14, 13q4 and 20q11.		[129,130,134]
Recurrent mutations	JAK-STAT signaling pathway	*STAT3, STAT5b, JAK3,*		[21,135,136]
	RNA helicase family	*DDX3X*		[22]
	Tumor suppressors	*TP53, MGA*		[22,23,50]
	RAS-MAPK signaling pathway	*NOTCH3, EPHA1, PTPRQ, PTPRK, GNAQ*		[81,137]
	Apoptosis	*FAS*		[138,139]
	Epigenetic modifiers	*ARID1A, ASXL1, BCOR, KMT2D, MLL2, EP300*		[23,124,125]
Epigenetic alterations	Hyper methylation	Cell cycle regulators*: CDKN2A, CDKN2B, CDKN1A* Tumor suppressors*: BCL2L11* (*BIM*), *DAPK1*, *PTPN6* (*SHP1*), *TET2*, *SOCS6*, and *ASNS*.		[140] [141]
	Histone modifications	EZH2: histone methyltransferase, aberrant overexpressed in ENKTCL, leading to activation of NF-kB signaling pathway.		[142,143,144]
	mi-RNAs	Downregulated	miR-26a, miR-26b, miR-28-5, miR-101 and miR-363. De-regulated miR-146a: leading to inhibition of TRAF6, downregulating NF-kB signaling.	[144,145]
		Upregulated	miR-155 and miR21	[137,146]
Gene Overexpression	*Survivin:*	Induced by LMP1, EBV latent viral proteins		[147]
	*MYC:*	Upregulation possibly through LMP1 latent viral protein.		[81,148,149]
	*PD-L1:*	Overexpression of the cell death ligand favoring immune evasion		[150,151,152]
	*RUNX3:*	Mediated by *MYC*, resulting in decreased apoptosis and increase cell proliferation		[148]
	*AURKA:*	Increased cell proliferation		[153]
	*PDGFRA:*	Overexpression of PDGFRα but absence of genomic alteration		[154]
Other	CD38	Transmembrane protein associated with poor outcome		[155]

## Data Availability

No new data were created or analyzed in this study. Data sharing is not applicable to this article.
